# Babesial infection in the Madagascan flying fox, *Pteropus rufus* É. Geoffroy, 1803

**DOI:** 10.1186/s13071-019-3300-7

**Published:** 2019-01-23

**Authors:** Hafaliana C. Ranaivoson, Jean-Michel Héraud, Heidi K. Goethert, Sam R. Telford, Lydia Rabetafika, Cara E. Brook

**Affiliations:** 1 Virology Unit, Institut Pasteur de Madagascar, Antananarivo, Madagascar; 2 Zoology and Animal Biodiversity, Faculty of Sciences, University of Antananarivo, Antananarivo, Madagascar; 3 Department of Infectious Disease and Global Health, Cummings School of Veterinary Medicine, Tufts University, North Grafton, MA USA; 4 Department of Ecology and Evolutionary Biology, Princeton University, Princeton, NJ USA; 5 Department of Integrative Biology, University of California, Berkeley, Berkeley, CA USA

**Keywords:** *Babesia*, Babesiosis, Madagascar, Madagascan flying fox, *Pteropus rufus*, Pteropodidae

## Abstract

**Background:**

Babesiae are erythrocytic protozoans, which infect the red blood cells of vertebrate hosts to cause disease. Previous studies have described potentially pathogenic infections of *Babesia vesperuginis* in insectivorous bats in Europe, the Americas and Asia. To date, no babesial infections have been documented in the bats of Madagascar, or in any frugivorous bat species worldwide.

**Results:**

We used standard microscopy and conventional PCR to identify babesiae in blood from the endemic Madagascan flying fox (*Pteropus rufus*). Out of 203 *P. rufus* individuals captured between November 2013 and January 2016 and screened for erythrocytic parasites, nine adult males (4.43%) were infected with babesiae. Phylogenetic analysis of sequences obtained from positive samples indicates that they cluster in the *Babesia microti* clade, which typically infect felids, rodents, primates, and canids, but are distinct from *B. vesperuginis* previously described in bats. Statistical analysis of ecological trends in the data suggests that infections were most commonly observed in the rainy season and in older-age individuals. No pathological effects of infection on the host were documented; age-prevalence patterns indicated susceptible-infectious (SI) transmission dynamics characteristic of a non-immunizing persistent infection.

**Conclusions:**

To our knowledge, this study is the first report of any erythrocytic protozoan infecting Madagascan fruit bats and the first record of a babesial infection in a pteropodid fruit bat globally. Given the extent to which fruit bats have been implicated as reservoirs for emerging human pathogens, any new record of their parasite repertoire and transmission dynamics offers notable insights into our understanding of the ecology of emerging pathogens.

**Electronic supplementary material:**

The online version of this article (10.1186/s13071-019-3300-7) contains supplementary material, which is available to authorized users.

## Background

Babesiae are Apicomplexan parasites (order Piroplasmida; family Babesiidae) which infect the erythrocytes of birds and mammals [1]. Much like parasites of their sister order, Haemosporida (the causative agents of malaria), babesiae can induce disease, including anemia and haemoglobinuria, when replicating in the red blood cells of mammalian hosts [2]. Among these hosts, insectivorous bats infected with *Babesia vesperuginis* display elevated reticulocyte and white blood cell counts, lowered haemoglobin levels, and splenomegaly, as compared with uninfected individuals [3, 4]. Such reports are intriguing, given widespread debate in the literature as to the extent to which bats - the purportedly tolerant reservoirs for virulent zoonoses, such as Ebola, Nipah, and SARS [5] - experience pathology from any microparasitic infections [6]. Admittedly, most discussions of bat pathogen tolerance have been limited to Old World fruit bats [7, 8], distinct from the insectivorous bat hosts of *B. vesperuginis. Babesia vesperuginis* has been identified in bats across Europe [9–11], the Americas [12] and China [13, 14]; such widespread host and geographical ranges suggest, contrary to popular dogma, that infection-associated pathology is unlikely to be severe.

Currently, *B. vesperuginis* remains the only *Babesia* sp. categorically recovered from bat blood, although DNA from dog *Babesia* (*B. canis*) was found in fecal samples collected from insectivorous bats in Hungary [15] (these authors were unable to eliminate the possibility that parasite DNA was derived from bat ingestion of an arthropod vector). Typically, babesiae are vectored by ixodid ticks, which acquire and transmit the piroplasmid during blood meals [1], though some have speculated (without confirmation) that the bat-specific soft tick, *Argas vespertilionis*, could act as a *B. vesperuginis* vector [3]*.* Studies of babesial infections in captive baboon populations suggest between-host transmission in the absence of available tick vectors, indicating that direct horizontal and/or vertical transmission may also be possible [16].

To date, no babesial infections have been described in any frugivorous bat species globally, including flying foxes of the Old World Fruit Bat clade (family Pteropodidae). Descriptions of erythrocytic protozoa in pteropodid bats have been instead restricted to the order Haemosporida [17–19]. Previous work on the island of Madagascar has documented insectivorous bat infections with microfilaria and with several hemoprotozoa (leucocytozoans, haemosporidians, and trypanosoma), but authors noted the absence of any blood parasites in two endemic Madagascan fruit bats (*Eidolon dupreanum* and *Rousettus madagascariensis*) [20, 21]. No previous study has examined hemoprotozoal infections of the Madagascan flying fox, *Pteropus rufus*, the largest of the three endemic pteropodids inhabiting the island and the focus of our work. We present evidence of babesial infection in the blood of *P. rufus.* We aimed to (i) identify and (ii) characterize these babesiae morphologically, phylogenetically and ecologically.

## Methods

### Capture and specimen collection

**Fig. 1:**
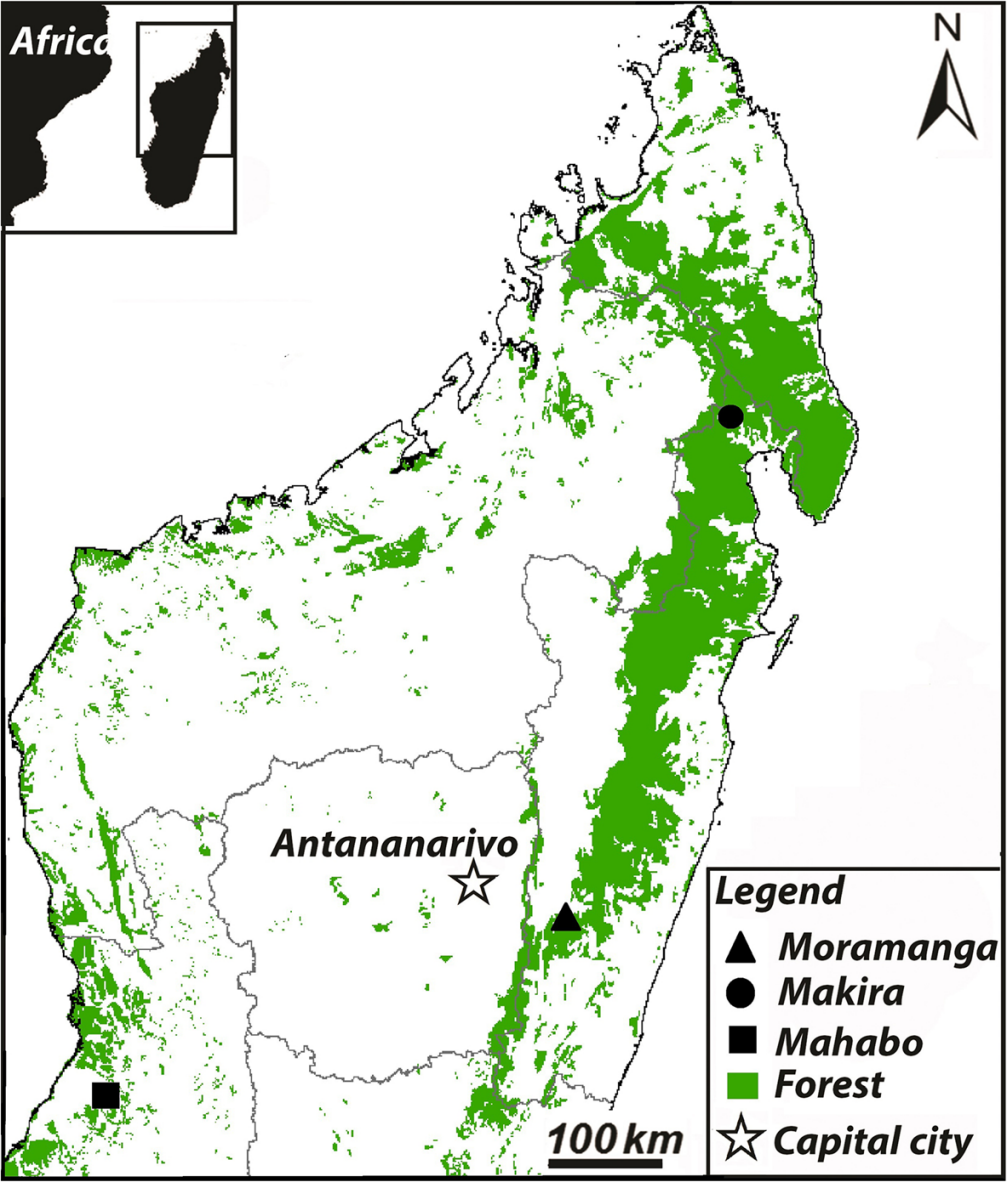
Map of Madagascar showing *Pteropus rufus* sample sites from November 2013 to January 2016. Green areas represent forest cover, black shapes represent site locations

We net-captured 203 tree-roosting *P. rufus* between November 2013 and January 2016 from three Madagascar localities: Mahabo (-20.39°S, 44.67°E, 57 m), Makira Natural Park (-15.11°S, 49.59°E*,* 226 m), and the District of Moramanga (-18.51°S, 48.17°E, 1000 m and -18.85°S, 48.06°E, 1034 m) (Fig. 1; Additional file 1: Table S1). Bats were captured in nine of twelve months of the year (except for April, June and October). Upon capture, bats were manually restrained, sexed, measured, weighed, and examined for ectoparasites. Between 0.4 to 1.0 ml of blood was drawn from the brachial vein of each captured individual, using a 1.0 ml insulin syringe. Blood specimens were centrifuged in the field, separated and stored in liquid nitrogen as blood clot and serum. Capillary tubes were used to collect blood from the puncture site for preparation of thin and thick-film blood smears. Blood smears were air-dried, fixed with absolute methanol and stored in a slide box containing desiccants. All captured bats were fed with sugar water post-processing and released alive at the site of capture the following evening.

A subset of adult *P. rufus* (*n* = 85) underwent anesthesia using a halothane vaporizer (4% halothane in oxygen at 0.7 l/min), during which time a lower left premolar tooth was extracted for aging purposes. Tooth samples were exported and processed histologically at Matson’s Laboratory (Missoula, Montana, USA), following previously published protocols [22, 23], to yield integer estimates of age *via cementum annuli* counts. Because fruit bats birth in synchronous annual pulses [24], we obtained more precise estimates of age by assuming a standard birth date (October 1) for all *P. rufus* captured in our study; we computed ages for pups less than one year (*cementum annuli =* 0) in the same way*.* We added the duration of time between capture and birth date to the integer estimate of age *via cementum annuli* to compute age to the nearest day for 144 *P. rufus* (80 male, 64 female)*.*

### Parasite identification by microscopy and PCR

All blood smears were Giemsa-stained and observed individually for 45 minutes each under a light microscope at ×1000 magnification, for primary detection of the parasite. Positive smears were subsequently re-scanned to identify all life stages of the parasite present (see ‘Morphological characterization of parasites’, below).

DNA was extracted from a 2 mm^3^ aliquot of each blood clot specimen, using the Macherey Nagel Dx Virus Kit (MACHERY-NAGEL, Düron, Germany), with slight deviations from the manufacturer’s instructions: each blood clot was homogenized in 120 μl of lysis buffer using a TissueLyser II (Qiagen, Hilden, Germany) at 25 Hz for 2 min. The lysate was centrifuged briefly, then 600 μl of lysis buffer and 20 μl of Proteinase K were added and incubated for 5 min at room temperature, followed by a second incubation at 70 °C for 1–2 h. After complete lysis, 600 μl of cold absolute methanol was added, mixed, and the solution was transferred to the column. The column was subsequently washed, and DNA was eluted with 50 μl of elution buffer.

In order to detect piroplasmid DNA, we first carried out PCR on all extracted DNA, using the published primers, PIROA/PIROB, following the authors’ protocol [25]*.* These pairs of primers amplify a 408 to 437 bp fragment from the *18S* rRNA gene of *Babesia* spp. and *Theileria* spp. Amplified DNA from *Babesia*-positive specimens was submitted for sequencing at Genewiz ltd. (Takeley, UK). Obtained sequences were compared to those available in the web-based database, blastn (http://blast.ncbi.nlm.nih.gov/Blast.cgi) for primary confirmation.

To improve phylogenetic inference, we next designed a second set of primers, Bab_F_Rufus (5'-TGA CAC AGG GAG GTA GTG AC-3') and Bab_R_Rufus (5'-TAG ACG AAT CTA AGC CCT CC-3'), which amplified a larger region (1200 bp) of the *18S* rRNA gene of *Babesia* spp. We carried out DNA amplification with these new primers in a reaction mixture containing 4 μl of DNA mixed with 1× of green GoTaq Flexi Buffer, 2 mM of MgCl_2_, 200 μM of each dNTP, 0.15 μM of each primer, and 1.25 U of GoTaq DNA polymerase (Promega Corporation, Madison, USA), and nuclease-free water. Thermal cycling was performed on a T3000 thermocycler (Biometra, Goettingen, Germany) under the following conditions: one cycle of 94 °C for 3 min followed by 35 cycles of: 94 °C for 45 s, 58 °C for 60 s, and 72 °C for 90 s. A final elongation was carried out at 72 °C for 10 min. A negative (nuclease free water) and positive control were included in each round of PCR. PCR products were visualized on a 1.5% agarose gel.

### Morphological characterization of parasites

Upon re-examination of parasite-positive blood smears, photographs of the parasite were taken with a Bresser MikroCam 5.0 MP digital camera (BRESSER UK LTD., UK) attached to the microscope. The included software, MikroCam lab, was used for analysis and measurement, after calibration of each objective with a micrometer glass slide. In total, we measured the diameter of 150 parasites in the trophozoite life stage. Parasites for measurement were randomly selected from the two blood smears which yielded the clearest and most accurate images under microscopy (AMBK_046, AMBK_047; Additional file 2: Table S2); all other smears were too blurry for image analysis.

Parasite load was estimated for all positive blood smears in which parasites were identified within the monolayer section of the smear (*n* = 5; estimation of parasitemia is impossible in thick, multi-layer smears). We calculated parasite load as the percentage of infected red blood cells (RBC) identified over 200 microscope fields at ×1000 magnification. To obtain this metric, we first estimated total RBC count by calculating the mean RBC count per microscope field from five randomly-selected fields within each smear monolayer, then multiplied that mean across the 200 observed fields. Parasitemia was obtained by dividing the total number of infected RBCs identified across all 200 fields by the estimated total RBC count, and multiplying by 100.

### Phylogenetic characterization of parasites

All specimens that showed a positive band at the expected size (1200 bp) upon gel electrophoresis after PCR with targeted primers (Bab_F_Rufus and Bab_R_Rufus) were sequenced on both strands (Genewiz ltd., Takeley, UK). We ran a database search using the BLAST web server (http://blast.ncbi.nlm.nih.gov/Blast.cgi) to identify *Babesia* spp. obtained from sequencing of positive samples. Sequences from all *Babesia* spp. identified were aligned with 27 full-length sequences obtained from representative *Babesia* and *Theileria* spp. in all piroplasmid subclades [1, 26–28], as well as with five partial sequences from closely-related lemur, cat, and baboon *Babesia* spp. and two partial sequences from the bat-infecting *B. vesperuginis.* Sequences were aligned under default parameters in the program MUSCLE [29], using *Plasmodium falciparum* as the outgroup, then trimmed to 572 bp to reflect the length of the shortest partial sequence in the dataset (from *B. vesperuginis*). Subsequent evolutionary analyses were conducted in MEGA7 [30]: we identified the Tamura 3-parameter with Gamma distribution and invariable sites (TN92+G+I) model as the best-fit for the data (*via* BIC score) and constructed the corresponding phylogenetic tree using a Maximum Likelihood approach. Initial tree(s) for the heuristic search were obtained automatically by applying Neighbor-Join and BioNJ algorithms to a matrix of pairwise distances estimated using the Maximum Composite Likelihood (MCL) approach, and then selecting the topology with superior log likelihood value. The resulting phylogenetic tree was inferred *via* complete gap deletion, for a total of 380 positions in the final database [31, 32].

### Ecological characterization of parasites

#### Seasonality of infection

We next sought to identify broad ecological trends in babesial infection of *P. rufus* across our longitudinal study. To this end, we assessed the relationship between site, season of capture, and *Babesia* infection status (positive/negative by PCR), using a generalized additive model (GAM) computed in the *mgcv* package in R [33]. We fixed infection status as the binomial response variable, determined *via* the fixed predictor variable of site and incorporating three site-specific smoothing terms based on the month (January-December) of bat capture. Because we were interested in broad seasonal variation, we fixed the smoothing term *k* at 4 and adopted a cyclic smoothing spline to force continuity from the end of one year to the beginning of the next (Additional file 3: Text S1). We interpreted statistically significant site-specific smoothing terms as evidence of seasonality in infection prevalence for the parasite.

#### Effects on host health

Given previous records of pathologic *B. vesperuginis* infections in other bat hosts [3], we next sought to identify any health impacts of infection with *P. rufus* babesiae. To this end, we first tested the association between standardized body mass (the response variable) for all adult male bats in our dataset and two predictor variables: forearm length (a measure of body size) and infection status (positive/negative by PCR), using a simple linear regression model (Additional file 3: Text S2). We next computed a measure of body condition by calculating the residual of each bat’s observed standardized mass from the predicted mass of the forearm: standardized mass regression line, recovering positive residuals for bats with higher-than-expected masses for a given forearm length and negative residuals for bats with lower-than-expected masses. We assessed these relationships using a one-sided Wilcoxon rank sum test with continuity correction which compared residuals across the two infection groups: babesiae-negative bats (*n* = 58) versus babesiae-positive bats (*n* = 9) (Additional file 3: Text S2).

We next carried out baseline relative white blood cell (WBC) counts on a subset of blood smears, to compare the physiological environment of babesiae-positive *versus* babesiae-negative hosts (Additional file 3: Text S3; Additional file 4: Table S8). To control for effects of site, season, age, and sex, we restricted negative blood smears for comparison to those obtained from the same sex, age class, month, and sites from which positives were recovered. From this sub-sample (*n* = 33; Additional file 1: Table S1), we randomly selected 14 infection-negative slides for comparison with the seven positive slides for which WBC counts were possible (Additional file 3: Text S3). After WBC counts were conducted, we compared the mean leukocyte density per microscope field across our two infection categories, *via* Welch’s two-sample, two-sided t-test.

#### Age-prevalence and force of infection

Finally, we assessed trends in age-structured *Babesia* prevalence in our system. Because we recovered no positive females during our sampling, we restricted this analysis to aged male *P. rufus* only (*n* = 80).We first tested the association between age and infection status, using a simple generalized linear model in the binomial family, with infection status (positive/negative by PCR) as the response variable and age (a continuous variable) as the predictor (Additional file 3: Text S4). To confirm that observed patterns in age-biased infection were not simply the result of age bias in body mass or condition, we additionally tested the relationship between age and standardized mass and age and forearm: standardized mass residual, using a linear model; tests were applied to the subset of aged adult male *P. rufus* only (*n* = 50; Additional file 3: Text S4).

We next sought to quantify the age-specific force of infection (FOI), by fitting a susceptible-infectious (SI) transmission model, indicative of a non-immunizing persistent infection, to our age-prevalence data. For this analysis, we followed previously described methods, assuming lifelong infection, negligible infection-induced mortality, and perfect sensitivity in our detection of babesial infections [34–37]. Because prevalence was low across our dataset, we modeled all male *P. rufus* data cumulatively and did not attempt to fit season- or site-specific variations in FOI. We considered more complex model structures incorporating multiple age-specific FOIs and evaluated each parameter addition via comparison of the Akaike’s information criterion (AIC) value produced from each model’s fit to the data (Additional file 3: Text S4). We computed 95% confidence intervals around all FOI estimates by standard error, as determined from the root square of the diagonal elements of the inverse of the Hessian matrix recovered from parameter optimization (Additional file 3: Text S4).

## Results

### Parasite identification, by microscopy and PCR

Nine of 203 (4.43%) *P. rufus* bats (all adult males) were positive for babesial infection by PCR, using both sets of primers described; seven of 203 (3.45%) were positive by initial microscopy (Table 1). We failed to detect infection by microscopy in two PCR-positive samples (ID: MAKR_011, from Makira; ID: AMBK_083 from Moramanga) in the initial round of 45 minute observations; however, upon subsequent re-examination of the slides following molecular detection, we identified a few small ring stages of the parasite in the head region (thick layer) of the original slides. While our research team successfully collected and identified an abundance of ectoparasites from other Malagasy fruit bat species during these sampling sessions, no ectoparasites were recovered from *P. rufus* bats [38]*.*

**Table 1:** Prevalence of babesial infection in *Pteropus rufus* across discrete sampling events

Site	Mid-date of sampling event	Sex	Prevalence (%) (*n* positive^a^/ *n* tested)
Mahabo	7-19-2014	F	0 (0/12)
		M	0 (0/7)
Makira	11-8-2014	F	0 (0/2)
		M	0 (0/1)
	7-16-2015	F	0 (0/6)
		M	16.67 (1/6)
Moramanga^b^	11-19-2013	F	0 (0/16)
		M	0 (0/15)
	2-22-2014	F	0 (0/1)
		M	0 (0/2)
	9-12-2014	F	0 (0/5)
		M	0 (0/8)
	12-9-2014	F	0 (0/28)
		M	23.53 (4/17)
	3-22-2015	F	0 (0/5)
		M	0 (0/22)
	5-19-2015	F	0 (0/2)
		M	0 (0/9)
	1-21-2016	F	0 (0/29)
		M	40 (4/10)

^a^Positives reported here indicate babesiae-positive by PCR

^b^Results were assessed statistically using GAMs [34] to query the relationship between babesial infection status, site, and month of sampling for male bats; data for females were not assessed since no positives were recovered (see Methods, for details). A significant monthly smoother, indicating a significant interaction between infection status and season, was recovered for male bats of the Moramanga site only (*P*-value = 0.0841) but not for Makira (*P* = 0.998) or Mahabo (*P* = 1), for which longitudinal data were scarce-to-nonexistent

*Abbreviations*: *F* female, *M* male, *n* number of animals

### Morphological characterization of parasites

**Fig. 2:**
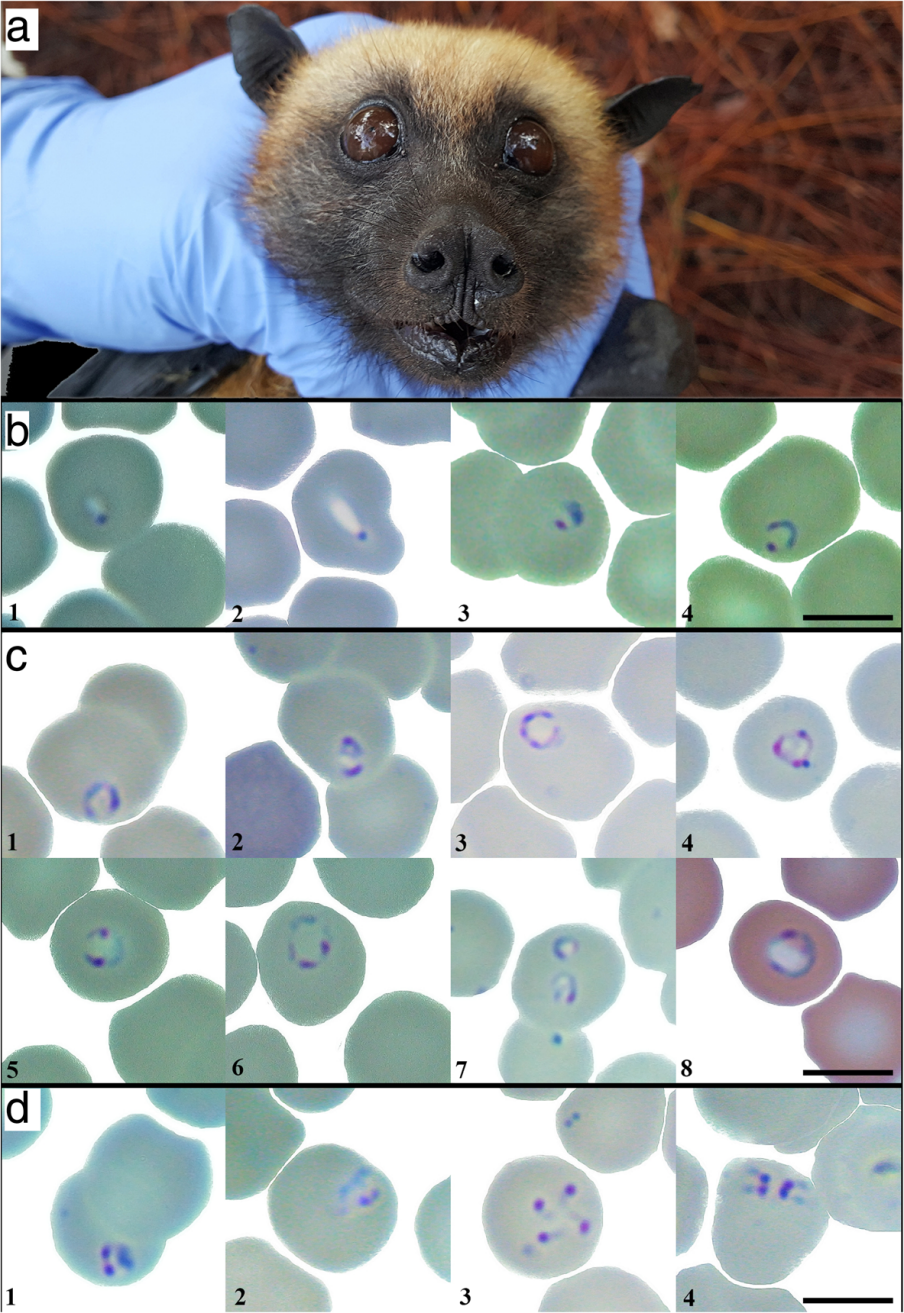
Babesial infection in red blood cells of *Pteropus rufus*. **a** The type-host, *Pteropus rufus*, a specimen from Ambakoana site. **b** Early stage trophozoites; b1, common forms for all positive samples b2, from ID AMBK_046, b3-4, from ID AMBK_047. **c** Unique forms for advanced stage trophozoites; c1-4, from ID AMBK_046, c5-7, from ID AMBK_047, c8, from ID AMBK_089. **d** Dividing forms from ID AMBK_046; d1-2, binary division, d3, four small merozoites, d4, typical ‘Maltese cross’ arrangement of the *Babesia* spp*.* (Giemsa staining, magnification of 1000×). *Scale-bars*: 5 μm

Microscopy revealed differences in the shape and size of babesiae identified within the RBC of *P. rufus*, though most samples appeared in ring-like form, consistent with the trophozoite life stage, when the young parasite is growing (Fig. 2b-c). In morphological observations of 150 randomly-selected trophozoites across our sample set, trophozoites appeared rounded-to-ovoid, with an average diameter of 1.87 ± 0.41 μm (mean ± SD; Additional file 2: Table S2). A variety of infected RBCs with single, double, and multiple dot chromatins were photographed, with single dot chromatins most common in early stage trophozoites (Fig. 2b) and multiple dot chromatins observed in advanced stage trophozoites (Fig. 2c).

In two samples from Moramanga (IDs: AMBK_046, AMBK_047), we observed the parasite multiplying within the red blood cell and documented multiple stages of the parasite life-cycle. Specifically, we documented binary fission (Fig. 2d:1, 2), during which advanced stage trophozoites divide to produce merozoites (Fig. 2d:3) that eventually align in the tetrad (‘Maltese cross’) formation characteristic of *B. microti*, which precedes erythrocytolosis and infection of new RBCs. No noticeable enlargement of infected red blood cells and no hemozoin pigments were observed in any infection.

We successfully estimated parasitemia for five of nine positive blood smears, though only 133 microscope fields were observable in a partially-damaged blood smear recovered from bat ID: AMBK_046 (in contrast to the standard 200 fields observed in the other four slides, see ‘Methods’). In the four smears which were not assessed, parasites were only found in the thick layer of the smear, making estimation of parasite load impossible. Of the five counted, we recovered the highest parasitemia (0.3%) in one sample from Moramanga (ID: AMBK_047); all other observed samples yielded parasitemias of less than 0.1%.

### Phylogenetic characterization of parasite

**Fig. 3:**
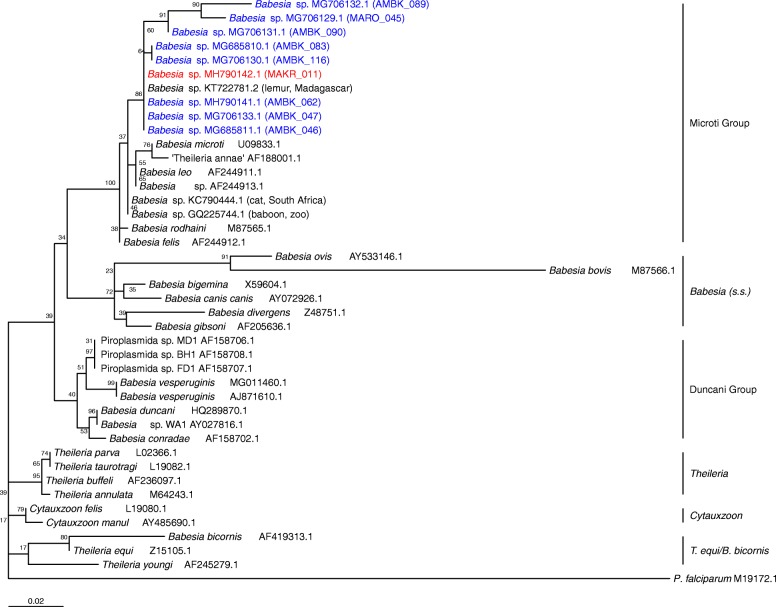
Maximum likelihood phylogeny of representative *Babesia* spp. genotypes obtained from complete and partial sequences of the *18S* rRNA gene. We inferred the evolutionary history of the piroplasmid clade from 42 sequences of the *18S* rRNA gene using inference based on a Maximum Likelihood approach and a Tamura 3-parameter model [33] with Gamma distribution and invariant rates (TN92+G+I). The base 27 piroplasmid sequences and the outgroup (*Plasmodium falciparum*) were full-length sequences, while the Madagascar *Pteropus rufus* sequences (9 total: those derived from Moramanga site shown in blue and Makira site in red) and closely-associated sequences from lemur (KT22781.2), South African cat (KC790444.1), and captive baboon (G]Q225744.1), as well as those from *B. vesperuginis* (MG011460.1 and AJ871610.1), were partial. The tree with the highest log-likelihood (-1610.95) is shown, with subclade names indicated to the right of the phylogeny. The tree is drawn to scale, with branch lengths measured in the number of nucleotide substitutions per site; all positions containing gaps and missing data were eliminated. There were a total of 572 positions in the initial alignment and 380 positions in the final dataset

In total, we recovered nine sequences of the *18S* rRNA gene of *Babesia* spp. from *P. rufus* blood sampled for this study: eight sequences from Moramanga and one sequence from Makira (GenBank: MG706129-MG706133, MH790141-MH790142 and MG685810-MG685811, indicated to the right of species names in Fig. 3). Using *Babesia microti* (XR_001160977) as a reference, new sequences correspond to the 477–1575 position of the full-length rRNA gene. All nine recovered sequences clustered together in the phylogenetic tree; the percentage of trees in which the associated taxa clustered together after 1000 bootstrap replications is indicated next to each branch (Fig. 3). We observed evidence of some genetic variation between recovered sequences (mean genetic distance ± SE: overall = 1.3 ± 0.02%; within Moramanga comparison = 1.4 ± 0.02%; mean Moramanga to one Makira sample = 0.08 ± 0.01%). In particular, sequences MG706129 and MG706132 were unique from the rest of the clade, showing, respectively, 937/970 bp (97%) and 970/1003 bp (97%) identity to the other seven sequences, which were near-identical. MG706129 and MG706132 were further unique from one another, showing 937/961 bp (98%) identity when compared internally.

BLAST of the *18S* rRNA partial sequences from *Babesia* spp. ex *P. rufus* matched most closely with a *Babesia* sp. sequence previously reported from Madagascan lemurs (KT722781.2; [39]), with 934/942 bp (99.15%) identity. Sequences showed 936/966 bp (97.89%) identity to a baboon *Babesia* sp*.* from a captive colony in the USA (GQ225744.1; [40]) and 935/970 bp (96.39%) identity to a *Babesia leo-*like species recovered from a domestic cat in South Africa (KC790444.1). Intriguingly, Madagascan flying fox *Babesia* spp. sequences demonstrated only 457/472 bp (88.70%) identity to previously described bat-infecting *B. vesperuginis* (AJ871610.1 [9]). Phylogenetic analysis (Fig. 3) suggests that observed Madagascan flying fox *Babesia* spp*.* fall within the monophyletic clade encompassing small-sized feline, rodent, primate and canid babesiae (the so-called ‘Microti Group’[1, 26, 27, 41]), distinct from *B. vesperuginis* sequences which, by contrast, clustered with *Babesia* spp. of the ‘Duncani Group,’ or ‘Western clade’ [42]. Because only partial sequences of the *18S* rRNA gene are currently available for *B. vesperuginis*, as well as for *P. rufus* genotypes and their closest matches in GenBank, phylogenetic inference across all clades was limited to a 573 bp fragment only (Fig. 3).

### Ecological characterization of parasite

#### Seasonality of infection

Eight infected bats originated from Moramanga (the most heavily sampled site), one from Makira, and none from Mahabo (Table 1). All infected bats were captured during the wet season at both sites: November-February in Moramanga and April-August in Makira. In Mahabo, bats were only sampled during the region’s dry season (July). Generalized additive modeling indicated marginally significant seasonality in infection status for male bats from Moramanga, the only site for which we possessed longitudinal data (Table 1; Additional file 3: Text S1, *P* = 0.084 for GAM monthly smoother for Moramanga).

#### Effects on host health

**Fig. 4:**
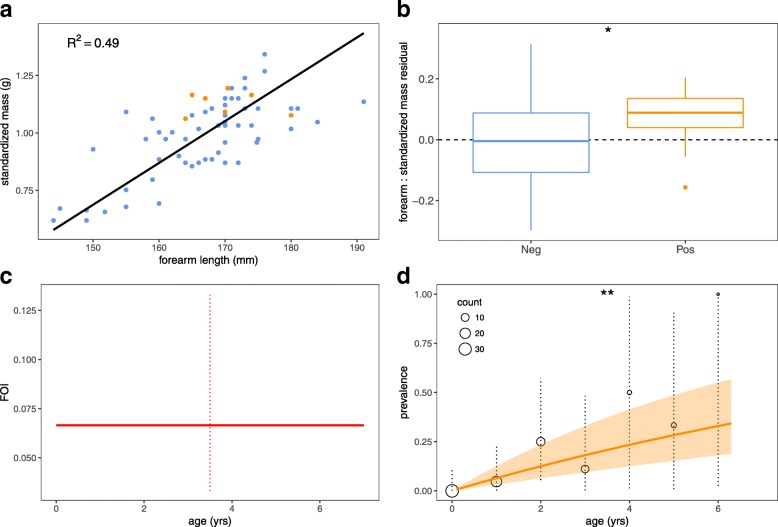
Ecological trends in babesial infection*.* **a** Standardized mass (in grams; y-axis) by forearm length (in mm; x-axis) for all adult male *P. rufus* fruit bats in our dataset (closed circles; infected = orange, uninfected = blue). Best-fit line from the fitted ‘standard major axis linear regression’ shown in black. *R*^2^ of the fitted model with predictor variables of forearm length and infection status (a factor) = 0.53. **b** Boxplot showing mean and interquartile range of forearm: standardized mass residual for babesiae-negative (neg., blue) and -positive (pos., orange) bats. Positive bats displayed a significantly higher residual by Wilcoxon rank sum test with continuity correction (W = 165, *P* = 0.039). **c** FOI estimated from best-fit SI model to age-prevalence data (mean = 0.0067; 95% CI by SE: 0.033–0.133). **d** Age-prevalence trends in babesial infection from raw data (open circles with 95% binomial exact CIs shown as dotted vertical lines) and fitted SI model (solid orange line = mean model output; translucent shading = 95% CI by SE)

Statistical analysis indicated that babesial infection, in addition to forearm length, was a significant (positive) predictor of standardized mass for male, adult-age *P. rufus* in our dataset (Fig. 4a; Additional file 3: Text S2, Table S3; *P* = 0.024). Bats positive for babesial infection were also found to have a significantly higher forearm: standardized mass residual than babesiae-negative bats (Fig. 4b; Additional file 3: Text S2; *P* = 0.040). Relative white blood cell counts carried out across a subset of babesiae-positive and -negative blood smears indicated no statistical difference in mean WBC count between positive and negative infection groups (Additional file 3: Text S3; Additional file 4: Table S8; *P* = 0.114).

#### Age-prevalence and force of infection

Generalized linear models indicated a statistically significant positive relationship between age and positive babesial infection status for male *P. rufus* (Additional file 3: Text S4; *P* = 0.004). We found no significant relationship between age and standardized mass or age and forearm: standardized mass residual, suggesting that age-prevalence trends do not result from age bias in mass or body condition but, rather, reflect a true relationship between age itself and the hazard of infection.

A single age-class susceptible-infectious (SI) model incorporating a constant force of infection across bat lifespan offered the best fit of all tested transmission models to age-prevalence data (Additional file 3: Text S4; AIC = 43.99). Because the prevalence of babesial infection across our entire dataset was very low, the estimated force of infection (the rate at which susceptible bats become infected) for this population was correspondingly low: 0.067 year^-1^ (Fig. 4c). Age-prevalence trends recovered from both data and fitted model demonstrated steadily increasing prevalence across the range of sampled age bins, consistent with assumptions of a lifelong, non-immunizing persistent infection (Fig. 4d).

## Discussion

To our knowledge, we report the first evidence of babesial infection in any pteropodid bat, using both microscopy and molecular methods. Specifically, we describe infection of novel *Babesia* spp. genotypes in blood from the Madagascan flying fox *Pteropus rufus.* Notably, these new *Babesia* spp. cluster phylogenetically within the *B. microti* clade, aligning most closely with a *Babesia* sp. lineage reported from Madagascan lemurs [39], rather than with the bat-specific *B. vesperuginis* [10]. We documented slight genetic variation among our recovered sequences, though no compelling evidence of clearly distinct genotypes. Mixed infections of multiple species within a single host are common in piroplasmids generally [43, 44] but were not identified here. Classically, *Babesia* spp. have been subdivided morphologically into small and large groups [45, 46], although more recent analyses have relied on molecular methods to reclassify *Babesia* spp. in four-to-six major phylogenetic subclades, including the ‘Microti Clade’ highlighted here [1, 26, 27, 41]. Morphological observations of *P. rufus* babesiae further support their placement in the ‘Microti Clade’, in which the typical trophozoite appears rounded, with a diameter of 1–2.5 μm [2] and with chromatins in a variety of single, double and multiple dot forms that multiply as the trophozoite advances in life stage [47]. The tetrad replication structure (‘Maltese cross’) observed in *P. rufus* babesiae is a characteristic feature of ‘Microti Clade’ infections [2].

Sequences derived from previously described bat-infecting *B. vesperuginis* appear to cluster within a whole different piroplasmid clade entirely (the ‘Duncani Group’) from that recovered for *P. rufus* babesiae. Two previous phylogenetic analyses, based on a small sequence fragment (624 bp) of the *18S* rRNA gene only [9, 10], instead placed *B. vesperuginis* in the ‘true *Babesia’* subclade, though more recent inference based on phylogenies of both *18S* rRNA and *cox1* genes, supports our results [48]. Ultimately, amplification of a more significant region of the *18S* rRNA gene for *B. vesperuginis* and accompanying morphological studies will be needed to clarify its placement. Regardless, it appears to be significantly different from the Madagascan flying fox *Babesia* spp. described here.

Most babesiae are vectored by ixodid ticks, which feed on a wide range of vertebrate taxa [45]. Ixodid ticks are present throughout Madagascar and known to infest a variety of host species, including lemurs in which babesial infections have been previously reported [49]. *Pteropus rufus* roost in trees that overlap lemur habitat [50], presenting opportunities for potential cross-species transmission by blood-feeding arthropods. Nonetheless, in 15 months of sampling, our field team failed to recover any ectoparasites from 203 captured *P. rufus*, despite successful collection of bat flies and fleas from other frugivorous bats sampled during this same period [38]. The arboreal lifestyle of *P. rufus* may subject the bats to environmental fluctuations and climatic instabilities that limit the extent of ectoparasite infestation, and it is possible that low prevalence ectoparasite infestations were simply missed. Ticks are known to switch among vertebrate hosts as they mature across advancing life stages [51], and their presence on any given species can be seasonal and brief. Previous studies of closely-related babesial infections in captive baboons also indicate that transmission and maintenance of these parasites may also be possible *via* horizontal or vertical mechanisms in the absence of available arthropod vectors [16].

As bats continue to garner attention for their links to emerging human zoonoses, a concrete understanding of the ecology of the diverse pathogen types that infect them - and the consequences of these infections for bat health - is becoming increasingly important. Our findings recapitulate much classic *Babesia* epidemiology from other systems. In particular, we uncovered evidence of seasonality in *Babesia* prevalence, with infection peaking during the wet season, a pattern consistent with the established literature [39, 52]. Additionally, we found that all bats identified as positive for babesial infection in our system were males, a sex-biased pattern also consistent with *Babesia* prevalence patterns reported in some other host systems [52–54]. This disparity may simply reflect a surface area effect in that larger-bodied individuals in this sexually size-dimorphic species [39, 55] have an elevated probability of ectoparasite infestation and corresponding infection risk, but could, likewise, result from true sex differences in immune function. Previous work has demonstrated that testosterone impairs both innate and acquired resistance to tick infestations in rodents and supports higher parasitemia and longer duration infections of *B. microti* in rodent hosts [56, 57]*.*

*Babesia vesperuginis* infections of insectivorous bats have gained notice for their potential capacity to cause disease in chiropteran hosts [3, 4] as, to date, few microparasites have been shown to induce any pathology at all in bats [6]. Our identification of the second-known lineage of bat-infecting babesiae therefore raises the question of its impact on bat health. The low infection rate recovered from our study precludes any sweeping inferences in this regard, though the nine babesiae-positive bats identified in our analysis appeared no less ostensibly ‘healthy’ than uninfected counterparts - and in fact, demonstrated higher average body mass for their size than did babesiae-negative individuals. As with male-biased infection, such results may simply reflect a surface area effect of higher probability of ectoparasite infestation and corresponding babesial infection in larger animals, but these findings could also indicate parasite preference for hosts better equipped to provide rich resource subsidies. In keeping with the lack of ostensible pathology, we observed no statistically significant differences in relative white blood cell counts between infected and uninfected hosts, although further sampling is needed to clarify whether babesial infection induces leukocytosis in *P. rufus*. The low number of infection-positive individuals recovered limited our inference in this regard. All told, categorical assessment of bat health was incomplete in our study, as no physiological assays were performed and no necropsies carried out on any sampled individuals (all bats were live-released following capture).

Congruent with patterns in sex- and body condition-biased infection, the majority of babesiae-positive *P. rufus* recovered in our study were older, yielding a broad pattern of increasing prevalence with age typical of that exhibited by any non-immunizing persistent infection. Age-prevalence data were best fit by a compartmental transmission model incorporating susceptible-infectious (SI) transmission dynamics, by which hosts are born susceptible, then subjected to a constant (albeit low) force of infection across the duration of their lifespans. As with body condition, our inference must be tempered by the rarity of positive samples recovered in our dataset; however, these patterns support our other observations of the lack of any pathological effect of infection on host health. Such findings are consistent with reports of persistent, low parasitemia babesial infections which can establish for years in other hosts, including mice [58, 59], cattle [60], and even humans [61, 62] - but counter to the established dogma of pathology associated with *B. vesperuginis* infections in insectivorous bats [3]. Further research will be needed to clarify the true extent of pathogenicity for these parasites and determine whether these discrepancies, if genuine, reflect differences in the two different babesiae, their hosts (i.e. fruit bats *versus* insectivorous bats), or the timing of the parasite life-cycle during which sampling took place. In babesial infections of other hosts, long-term persistent infections can establish after control and survival of acute pathology [2, 63], meaning that sampling during only part of the parasite life-cycle could yield biased interpretations of virulence.

In other systems, babesiae have been identified as zoonotic agents, emerging from cattle (*B. divergens* [64]) and rodent (*B. microti* [65]) reservoirs to cause disease in humans. As ixodid ticks are known to feed on both wildlife and humans in Madagascar [49, 66], zoonotic emergence of bat-infecting baesiae is theoretically possible. Phylogenetic clustering of recovered sequences of *Babesia* spp. from *P. rufus* with primate *Babesia* spp. supports the potential for cross-species transmission, although the low prevalence and parasitemia observed in babesial infections of the Madagascan flying fox make *P. rufus* an unlikely candidate reservoir for human *Babesia.* Nonetheless, as previous reports indicate the presence of potentially zoonotic viral pathogens circulating in the Malagasy fruit bat community [67, 68], any heightened understanding of the transmission dynamics and parasite repertoire of these robust pathogen hosts is relevant to efforts to safeguard human health.

## Conclusions

To our knowledge, this study reports the first global record of a babesial infection in an Old World fruit bat of the family Pteropodidae, as well as the first record of babesial infection in any bat in Madagascar. Comparisons of the *18S* rRNA gene of parasites recovered from this study indicate that the new *Babesia* spp. sequences are distinct from previously identified bat-infecting *B. vesperuginis,* instead aligning most closely with sequences derived from Madagascan lemurs and other parasites in the *B. microti* clade. Our findings offer unique insights into the landscape of microparasitic infection for fruit bat reservoirs for emerging infectious disease.

## Additional files

Additional file 1: **Table S1.** Raw data used in analyses, including: sampleID, site/date of capture, species, sex, age class, age, mass, forearm length, babesial infection status (0/1) and white blood cell density per microscope field (subset only) for all *P. rufus* sampled in this study. (CSV 16 kb)

Additional file 2: **Table S2.** Raw measurement data from 150 randomly sampled trophozoites observed from blood smears of sample IDs: AMBK_046 and AMBK_047. (CSV 2 kb)

Additional file 3: **Text S1.** GAM constructions for infection seasonality. **Table S3.** Output from GAM significance tests. **Figure S1.** Monthly GAM smoother, babesial infection in Moramanga. **Text S2.** Relationship between body condition and infection status. **Table S4.** Linear regression output of forearm: standardized mass residual. **Text S3.** Comparing WBC across infection status. **Figure S2.** Mean leukocyte density per microscope field across babesial infection status. **Text S4.** Age-prevalence and the force of infection. **Table S5.** Generalized linear model output, infection status by age. **Table S6.** Comparison of age-structured SI model fits. **Table S7.** Parasitemia in babesiae-positive blood smears. (PDF 270 kb)

Additional file 4: **Table S8.** Raw white blood cell (WBC) counts for 15 randomly sampled microscope fields across a subset of babesiae-positive and negative bats. Note that only 9 microscope fields are reported for sample ID MARO_052, as all other fields viewed in the initial 50 were disregarded because no leukocytes were identified (Additional file 3: Text S3). (CSV 10 kb)
