# Supplementary material for: Babesial infection in the Madagascan flying fox, Pteropus rufus É. Geoffroy, 1803
Source: Parasit Vectors. 2019 Jan 23;12:51. doi: 10.1186/s13071-019-3300-7 (PMC6343336; doi:10.1186/s13071-019-3300-7)
Supplement: Supplementary file 3 — Text S1. GAM constructions for infection seasonality. Table S3. Output from GAM significance tests. Figure S1. Monthly GAM smoother, babesial infection in Moramanga. Text S2. Relationship between body condition and infection status. Table S4. Linear regression output of forearm: standardized mass residual. Text S3. Comparing WBC across infection status. Figure S2. Mean leukocyte density per microscope field across babesial infection status. Text S4. Age-prevalence and the force of infection. Table S5. Generalized linear model output, infection status by age. Table S6. Comparison of age-structured SI model fits. Table S7. Parasitemia in babesiae-positive blood smears. (PDF 270 kb) [file 13071_2019_3300_MOESM3_ESM.pdf]

Additional File 3: Appendix 1.

Text S1: GAM constructions for infection seasonality

We used a generalized additive model (GAM) in the mgcv package in R [26] to assess the relationship between babesial infection status (a binomial response variable), sampling site, and month of capture. We applied our GAM to male bat data only (available in Supplementary Data 1, Additional File 1), since no positive samples were recovered from females.

We fixed the smoothing term k at a value of 4 to recover quarterly seasonality and used a cyclic smoothing spline to force continuity at the December/January transition.

Our GAM took on the following form:

```
m1 = gam(PCR_pos_Babesia~site +
            s(month,  by = as.numeric(site=="Moramanga"), k=4, bs = "cc") +
            s(month,  by = as.numeric(site=="Mahabo"), k=4, bs = "cc") +
            s(month,  by = as.numeric(site=="Makira"), k=4, bs = "cc"),
            family = "binomial", data=bab.dat.M)
```

We recovered a marginally significant (p<.1) positive smoothing term for the Moramanga site only, indicating seasonality in babesial infection status at the one site for which we possessed extensive longitudinal data (seven discrete sampling events, see Table 1 of main text).

Smoothing term results from fitted GAM are summarized in Table S3.

Table S3. Output from GAM significance tests

smoother	edf	chi-squared statistic	p-value
month, by = Moramanga	1.834e+00	5.447	0.084*
month, by = Mahabo	-8.431e-17	0	1
month, by = Makira	2.416e-05	0	0.9984

significance via p-value < .1*, .01**, .001***

The seasonal smoother for the Moramanga site took on the following form (Fig. S1), indicating elevated probability of positive babesial infection status during the wet seasons for this site (months November-February).

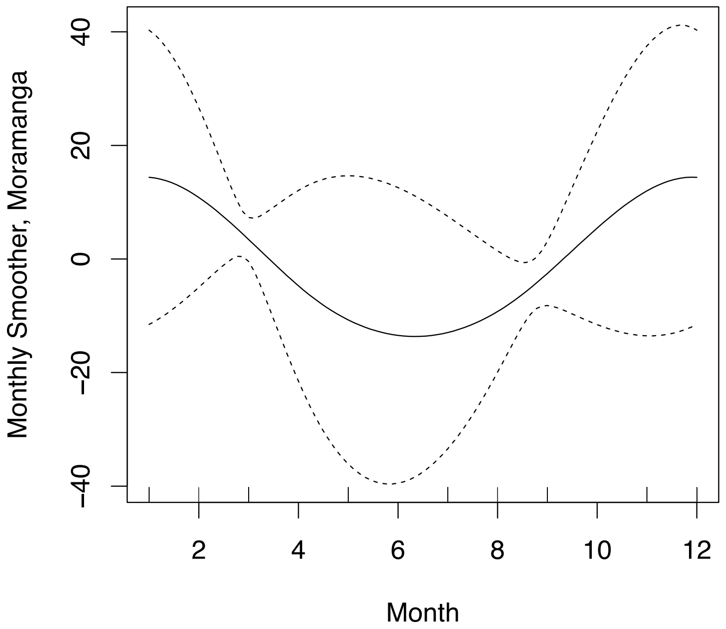

Figure S1. Monthly GAM smoother, babesial infection in Moramanga
The smoother from the fitted GAM (Text S1; Table S3) for the Moramanga site, indicating seasonality in babesial infection for male *P. rufus* bats in our dataset. The y-axis gives the extent to which the probability of positive/negative infection is modulated by month of year, as indicated on the x-axis. Months are represented as numerals, from January (1) to December (12). 95% confidence intervals are shown as dotted lines around the mean. The smoothing term, k, was fixed at 4, and we used a cyclic smoothing spline which forces the smoother to move continuously from December (12) to January (1).

Text S2: Relationship Between Body Condition and Infection Status

To assess the relationship between body condition and infection status with babesiae (+/- by PCR), we first sought to establish a standardized measure of below/above body condition for adult male fruit bats in our dataset, using the residual of the forearm:mass regression. We ignored juvenile bats in this analysis, since both size (forearm length) and mass are more likely to correlate with age than with condition in animals that are not yet full grown. Additionally, since *Pteropus rufus* are size-dimorphic by sex (males larger than females), we sought to compare males separately from females. Ultimately, since no female bats were found to be infected with babesiae in this analysis, we examined this relationship only in adult male individuals (N= 67) in our dataset.

We first assumed that larger-bodied adult male bats (with longer forearm lengths) would be, on average, more massive than smaller-bodied males (with shorter forearm lengths). To express this relationship, we first standardized masses by dividing the masses of all adult males in the dataset by their mean (677.9g) to enable later comparisons with other species or sexes. We then plotted forearm length as the explanatory variable against the response variable of standardized mass and fit a "standard major axis" type 2 linear regression line to the data points (Fig. 4a, main text, reproduced here for convenience). Type 2 linear regression assumes variation and error in measurements for both x and y-variables [66]. We determined predicted values of standardized mass for all forearm lengths measured in our analysis using the output slope (m = .0182) and the y-intercept (y = -2.043) from the fitted type 2 regression model, then plotted these predictions as a best fit line atop raw data in Fig. 4a.

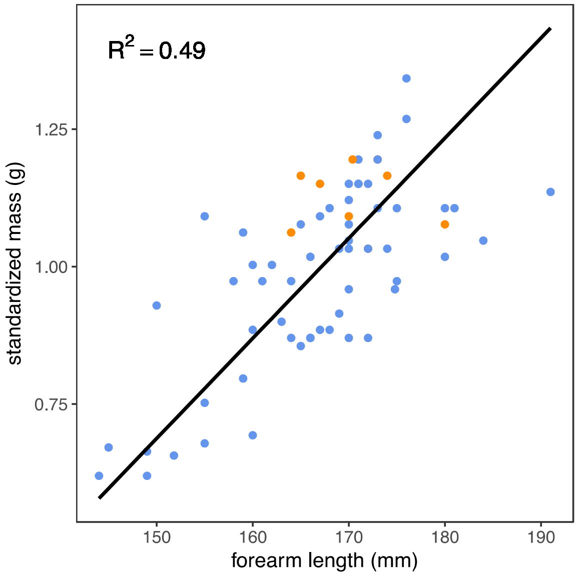

Fig. 4a. Forearm: mass standardized residual for adult male *Pteropus rufus*.
Forearm length (in mm; x-axis) plotted against standardized mass (in grams; y-axis) for all adult male *P. rufus* fruit bats in our dataset (closed circles; infected=orange, uninfected=blue). Best-fit line from the fitted 'standard major axis linear regression' for these bats is shown in black. R^2 of fitted model = .53.

We then assessed the relationship between infection status and body condition, using a simple linear model. We set standardized mass as the response variable to the predictors of forearm length and infection status (a factor), using the following R code construction:

```
m1 = lm(std_mass~forearm_length + factor(infection_status), data = dat.bab.BMI)
```

A summary of the fitted model indicated that both forearm length and infection status were significant predictors of standardized body mass (Fstat = 36.12 on 2 and 64 DF, p-value: 3.158e-11; R^2 = .53).

Table S4. Linear regression output of forearm: standardized mass residual

Predictor	slope [lci – uci][†]	t-value	p-value
forearm length	0.012 [0.009 - 0.015]	7.74	9.33e-11 ***
infection status	0.097 [0.015 - 0.179]	2.32	0.023563 *

significance via p-value < .1*, .01**, .001***
[†]lci and uci indicate lower and upper confidence intervals by standard error: mean±1.96*SE

We next determined the residual of the forearm: mass relationship for each bat in our dataset by subtracting the observed masses (Fig. 4a: closed circles) from the predicted values (Fig. 4a: black line) for each forearm length. Bats which were heavier than predicted for their body size thus recovered positive residuals, while bats thinner than predicted for their body size recovered negative residuals.

Finally, now that each individual bat had an established forearm: mass residual, we split the dataset into babesiae-negative individuals (N = 58) and babesiae-positive individuals (N = 9) and compared their residuals across the two infection groups (babesiae-negative bats, N=58; versus babesiae-positive bats, N=9), using a one-sided Wilcoxon rank sum test with continuity correction. Given the physical presentation of the data (Fig. 4b, main text), we queried the null hypothesis that babesiae-positive bats had a higher forearm: standardized mass residual than babesiae-negative bats. Results were significant at W = 165, p-value = 0.03955.

Text S3: Comparing WBC Across Infection Status

Total leukocyte (white blood cell, WBC) counts were carried out on seven of nine babesiae-positive smears and a random subsampling (N=14) of negatives collected from adult males in the same three sites and months of year in which the positives were recovered (July in Makira and December/January in Moramanga). Fifty (50) microscope fields were examined at 400x magnification from the monolayer (thin film) section of each slide for these 21 individuals, and all visible leukocytes were counted. According to convention, we then disregarded all microscope fields in which no leukocytes were identified. To standardize effort, we randomly selected 15 fields per slide from the remaining pool (>0 observed WBCs) for comparison (though no analytical differences were observed between comparison of all counted fields versus this standardized subsample). The author who conducted the counts (HCR) was blind to the infection status of the sample at the time of counting.

In two of the nine babesiae-positive samples (AMBK_116 and MARO_045), only thick film (multilayer) slides were recovered, making WBC counts impossible. In a third, randomly-selected negative sample (MARO_052), leukocytes were only identified in nine out of the initially examined 50 microscope fields, yielding a smaller field number for comparison, after discounting all fields for which WBCs=0. Because of this, we compared mean leukocyte density per observation field in the two infection groups, rather than the total count. For MARO_052, this meant the total count was divided by nine; for all other samples, total count was divided by 15.

We plot the mean leukocyte density per observation field across babesiae-positive and -negative infection groups in Supplementary Fig. 2. We compared mean leukocyte density per observation field across babesiae-positive and negative infection groups, using a Welch's Two Sample, Two-Sided T-test in R, querying the two-sided alternative hypothesis that infected bats had a different mean WBC count than uninfecteds. Results were not significant, indicating that we can see identify no statistical difference in mean WBC count between babesiae-positive and -negative adult *P. rufus* males ($t = 1.7772$, $df = 7.8824$, p-value = 0.114). We additionally queried the one-sided alternative hypothesis that babesiae-negative samples had a lower mean WBC density per microcope field than positives. This test was borderline significant ($t = 1.7772$, $df = 7.8824$, p-value = 0.057*). Further data will be needed to assess whether there is any significant associate between babesial infection and leukocytosis for *P. rufus.*

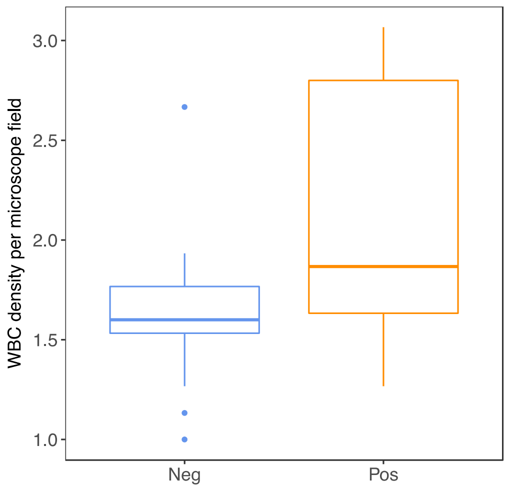

Figure S2. Mean leukocyte density per microscope field across babesial infection status. Boxplot showing mean and interquartile range of leukocyte (White Blood Cell, WBC) density per observed microscope field for babesiae-negative (blue, N= 7) and -positive (orange, N=14), bats. Blood smears examined for negative bats were randomly sub-sampled from adult males caught in the same months at the same sites from which positives were recovered. We observed no statistically significant difference between leukocyte density across infection groups (Welch's two sample t-test: t = 1.7772, df = 7.8824, p-value = 0.114).

Text S4: Age-Prevalence and the Force of Infection

We first assessed the relationship between age and infection status, using a simple generalized linear model applied to the subset of male bat data for which we possessed estimates of age (N=80). The model took on the following structure (with age as a continuous variable and infection status as a factor):

m2 = glm(infection_status~age, family= “binomial)

Results of the model indicated a positive relationship between positive infection with babesiae and elevated age (Table S5).

Table S5. Generalized linear model output, infection status by age

Predictor	slope [lci – uci] †	z-value	p-value
age	0.713 [0.234-1.191]	2.92	0.00352**

significance via p-value < .1*, .01**, .001***
†lci and uci indicate lower and upper confidence intervals by standard error: mean±1.96*SE

To confirm that patterns in age-biased infection were not simply the result of age bias in body mass or condition, we additionally tested the relationship between standardized mass and age and forearm: standardized mass residual using standard linear models, which took on the following form:

m3 = lm(standardized_mass ~age, data=bab.BMI.age)
Summary: Adjusted R-squared: 0.02902; F-statistic: 2.464 on 1 and 48 DF, p-value: 0.123

m4 = lm(residual_forearm_standardized_mass ~age, data=bab.BMI.age)
Summary: Adjusted R-squared: -0.005625; F-statistic: 0.7259 on 1 and 48 DF, p-value: 0.3984

Age was not a significant predictor in either model, suggesting that age-biased patterns in infection prevalence do not result from an alternative, underlying variable but, rather, from age itself.

In light of these findings, we next computed the age-specific force of infection (FOI, or λ), by fitting a susceptible-infectious (SI) catalytic model to our age-prevalence data, using methods that have been previously described [33–36, 67]. For convenience, we summarize these methods in the Appendix here.

Following Long et al. 2010 [35], we organized our data subset into two columns: 'age', which listed the age of the individual and 'sick' which listed a 0 for uninfected individuals and 1 for infected (and infectious) individuals (as determined by *Babesia* spp. PCR; see main text for details). Following Pomeroy et al. 2015 [67] and Brook et al. 2017 [33], we considered a two disease state system in which bat hosts were classified into Susceptible and Infectious proportions of the total population, such that S + I = 1.

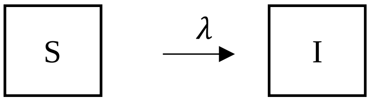

The force of infection (FOI, or λ) gives the rate at which susceptibles become infected and is equal to the pathogen transmission rate multiplied by the proportion of the population infectious at a given time. Again, following Pomeroy et al. 2015 [67] and Brook et al. 2017 [33], we assume an age-specific force of infection, represented by $\lambda(a)$, such that:

$$\frac{dI(a)}{da} = \lambda(a)\big(1 - I(a)\big)$$

Solving for $I(a)$ yields:

$$I(a) = 1 + C * exp\left[-\int_0^a \lambda(a)da\right]$$

Consistent with previously published work [33, 67], we make the assumption that all individuals are susceptible at birth, such that $I(0) = 0$, by which $C = -1$. From this, our equation further reduces to:

$$I(a) = 1 - exp\left[-\int_0^a \lambda(a)da\right]$$

where λ(a) is the age-dependent force of infection. Following Long et al. 2010 [35], we assume a constant FOI for pre-determined age intervals. When the FOI is assumed to be piece-wise constant across k age classes and each segment has a starting age l_a and duration d_k, the integrand in the preceding equation for an individual of an age within the k'th age class is given by:

$$\int_0^a \lambda(a)da = \lambda_k(a - l_k) + \sum_{a=0}^{k-1} \lambda_a\, d_a$$

Adopting R-code from the supplementary material of Long et al. 2010 [35] and Brook et al. 2017 [33], we estimate the age-class specific forces of infection $\lambda(a)$ by minimizing the negative log-likelihood with the R function dbinom(). First, we use a general function to return the negative log-likelihood from the model when compared with the data. The function inputs

are a series of starting values for the $\lambda(a)$ estimates (par), the dataset to be analyzed (data) and the lower cut-off for each age class in our model (cate). The function then follows as:

```
loglikpc=function(par,data, cate){
    dur=c(diff(cate), 0)
    ll=0
      for(a in 1:length(data$age)){
          dummy1=data$age[a]>cate
          dummy2 = data$age[a]>cate & !c(data$age[a]>cate[-1], FALSE)
          dummy1=c(data$age[a]>cate, FALSE)[-1]
          inte=sum(dur*exp(par)*dummy1)+ exp(par[dummy2])*(data$age[a]-cate[dummy2])
          p=1-exp(-inte)
          ll=ll+dbinom(data$sick[a],1,p,log=T) \}
    return(-ll)}
```

As described by Long et al. 2010 [35] and Brook et al. 2017 [33], the first line of the function calculates the duration of each age class, the second line sets the log-likelihood to zero, and lines 3-9 encompass a for-loop which calculates the hazard of infection for each individual in the dataset by integrating over the age classes already passed and the duration of time already spent in the current age class. Dummy variables are used to identify the preceding and current age class for each individual of age a. Line 9 gives the predicted hazard of infection for a given age (a) from the catalytic model, and line 10 evaluates the observed infection status for an individual of this age against the prediction using the binomial log-likelihood.

We can then use this function to estimate $\lambda(a)$ with the optim() function in R. For this, we bring in a vector of cut-off ages which determine the lower threshold of each age bin in our dataset (i.e. cate=c(0,.5) for a model incorporating two age-specific FOIs: one for neonates under six months and one for all other ages), provide some reasonable starting estimates for age-specific λ, and optimize $\lambda(a)$ using the quasi-Newton 'BFGS' method recommended in Bolker 2008 [68]. The optimization function takes on the following form:

```
out = optim(par=log(c(.5,.5), fn=loglikpc, cate= c(0,.5), method="BFGS", data=bab.age,
      control=list(trace=2, maxit=1000), hessian=TRUE)
```

The optimization outputs for the age-specific FOI estimates are given by out$par and the negative log-likelihood of the comparison of the data and the model prediction by out$value.

We can then compute the 95% confidence intervals around FOI estimates by standard error, as determined from the root square of the diagonal elements of the inverse of the Hessian matrix returned from optim(). We use the following R-code for this final computation:

```
FOI = exp(out$par)
hess <- solve(out$hessian)
SE <-sqrt(diag(hess))
uci<-exp(out$par+1.96*SE)
lci<-exp(out$par-1.96*SE)
FOI_CI <- data.frame(value=FOI, uci=uci, lci=lci)
```

Ultimately, given the sparseness of positive values in our dataset, we determined that a single age-class model, incorporating a constant FOI across the duration of the host's lifespan, offered the most justifiable fit to the data. We compared this model structure with a model structure incorporating two age classes, which allowed for variation in the FOI between young bats and adults (Table S6). We considered two cut-offs for the threshold age difference between young and adult-age bats: one at six months (dividing data into neonate and juvenile + adult age classes) and one at one year (dividing data into neonate + juvenile and adult-age classes). Since two age classes were not supported by this analysis, we did not evaluate models incorporating additional (i.e. three) age-specific estimates of FOI.

Note that we also compared the performance of the 'Nelder-Mead' optimizer when estimating multiple simultaneous parameters because it appeared to offer parameter estimates which better fit the data (Table S6). 'Nelder-Mead' is not supported for single parameter optimization. We summarize all model comparisons and report best fit parameters in Table S6.

Table S6: Comparison of Age-structured SI Model Fits

**# age classes**	**optimizer**	**age-bin** (years)	**FOI** (mean [lci-uci]†)	**negative log-likelihood**	**AIC**
**1**	**'BFGS'**	**(0+)**	**0.067 [0.033 - 0.133]**	**20.99**	**43.99***
2	'BFGS'	(0-.5)	.252 [0.126 - 0.504]	24.58	51.17
		(.5+)	9.79e-12 [0 - Inf]		
2	'BFGS'	(0-1)	.144 [0.072 - 0.289]	23.48	48.97
		(1+)	5.72e-08 [NA – NA]^		
2	'Nelder-Mead'	(0-.5)	7.719e-09 [0 – Inf]	20.32	42.64*
		(.5+)	.091 [0.045 - 0.181]		
2	'Nelder-Mead'	(0-1)	.144 [0.072 - 0.289]	23.48	48.97
		(1+)	4.19e-08 [NA - NA]^		

*Best fit models by AIC were compared via likelihood ratio test (2*ll(m2) - 2*ll(m1), where m2 is the less-restrictive model with more parameters):
2x(-20.32 + 20.99) = 1.34 chi-sq (corresponding p-val = 0.510, indicating support retained for the single-age class model only)
†lci and uci indicate lower and upper confidence intervals by standard error, as computed from the Hessian matrix (see Text S4, above)
^NAs produced when hessian recovered from optimization was not positive definite. These models were not supported regardless.

Ultimately, only a single-age class model, incorporating a constant force of infection across the bat's lifespan, was supported.

Table S7. Parasitemia in babesiae-positive blood smears

**Sample ID**	**Total Fields Surveyed**	**Mean # RBC/Field**	**Infected RBCs across all fields**	**Parasitemia** (% infected of total)
AMBK_046	133	383	42	0.082
AMBK_089	200	427	60	0.070
AMBK_047	200	386	231	0.299
AMBK_062	200	423	23	0.027
AMBK_090	200	360	8	0.011
